# Signaling Interactions in the Adrenal Cortex

**DOI:** 10.3389/fendo.2016.00017

**Published:** 2016-02-29

**Authors:** András Spät, László Hunyady, Gergő Szanda

**Affiliations:** ^1^Department of Physiology, Semmelweis University Medical School, Budapest, Hungary; ^2^Laboratory of Molecular Physiology, Hungarian Academy of Sciences, Budapest, Hungary

**Keywords:** cAMP, Ca^2+^ signal, mitochondria, aldosterone, angiotensin II, ACTH, potassium ion, cortisol

## Abstract

The major physiological stimuli of aldosterone secretion are angiotensin II (AII) and extracellular K^+^, whereas cortisol production is primarily regulated by corticotropin (ACTH) in fasciculata cells. AII triggers Ca^2+^ release from internal stores that is followed by store-operated and voltage-dependent Ca^2+^ entry, whereas K^+^-evoked depolarization activates voltage-dependent Ca^2+^ channels. ACTH acts primarily through the formation of cAMP and subsequent protein phosphorylation by protein kinase A. Both Ca^2+^ and cAMP facilitate the transfer of cholesterol to mitochondrial inner membrane. The cytosolic Ca^2+^ signal is transferred into the mitochondrial matrix and enhances pyridine nucleotide reduction. Increased formation of NADH results in increased ATP production, whereas that of NADPH supports steroid production. In reality, the control of adrenocortical function is a lot more sophisticated with second messengers crosstalking and mutually modifying each other’s pathways. Cytosolic Ca^2+^ and cGMP are both capable of modifying cAMP metabolism, while cAMP may enhance Ca^2+^ release and voltage-activated Ca^2+^ channel activity. Besides, mitochondrial Ca^2+^ signal brings about cAMP formation within the organelle and this further enhances aldosterone production. Maintained aldosterone and cortisol secretion are optimized by the concurrent actions of Ca^2+^ and cAMP, as exemplified by the apparent synergism of Ca^2+^ influx (inducing cAMP formation) and Ca^2+^ release during response to AII. Thus, cross-actions of parallel signal transducing pathways are not mere intracellular curiosities but rather substantial phenomena, which fine-tune the biological response. Our review focuses on these functionally relevant interactions between the Ca^2+^ and the cyclic nucleotide signal transducing pathways hitherto described in the adrenal cortex.

## Introduction

The adrenal cortex contains three zones, of which *glomerulosa* secretes the mineralocorticoid aldosterone, *fasciculata* synthesizes the glucocorticoid cortisol (or corticosterone in rodents), whereas the *reticularis* produces androgens [reviewed in Ref. (1)]. Aldosterone, acting on the distal nephron, augments Na^+^ reabsorption as well as K^+^ and H^+^ excretion. Through changes in sodium balance, it influences the extracellular fluid space and blood pressure, and its importance in cardiovascular, renal, and inflammatory diseases has also been recognized (2–4). Cortisol, among other things, controls intermediary metabolism, modulates immune responses, and is essential for the resistance of the organism to noxious stimuli. Adrenal androgens exert important anabolic effects in females and have substantial clinical significance in adrenal pathologies.

Sodium and/or fluid depletion, hemodynamic changes, and hyperkalemia stimulate aldosterone secretion. When fluid loss is severe, ACTH synergizes with angiotensin II (AII) in stimulating glomerulosa cells. During hypervolemia, atrial natriuretic peptide (ANP) inhibits aldosterone secretion [for reviews, see Ref. (5, 6)]. Cortisol production is governed by ACTH. The regulation of ACTH secretion and the signaling in zona *reticularis* (7) are beyond the scope of this review.

## Classical Signaling Pathways in the Adrenal Cortex

### Signaling Pathways in Glomerulosa Cells

The major signaling pathways of ACTH, K^+^, and AII, termed “classical” here have been described in several reviews [e.g., Ref. (5, 6, 8, 9)] and are only briefly summarized below.

ACTH binds to the melanocortin-type receptor MC2R, which activates adenylyl cyclase (AC) *via* the heterotrimeric G-protein G_s_ (10, 11), and subsequent cAMP formation activates protein kinase A (PKA). PKA then phosphorylates and induces the hormone-sensitive lipase (previously “cholesterol ester hydrolase”) (12) as well as the steroidogenic acute regulatory protein (StAR), the protein transporting cholesterol into the mitochondria (13, 14). As a result of these, the steroid precursor cholesterol is released from lipid droplets and transported to side-chain cleavage by CYP11A1, located in the inner mitochondrial membrane. This causes the stimulation of adrenal steroidogenesis.

Extracellular K^+^ and AII act by generating cytosolic Ca^2+^ signal. Depolarization induced by physiological elevations of [K^+^] activates T-type voltage-dependent Ca^2+^ channels the current of which was detected in rat (15–17), bovine (16, 18, 19), and human glomerulosa cells (20). Concomitant cell swelling evoked by K^+^ also enhances this T-type current (21, 22).

The unique sensitivity of glomerulosa cells to K^+^ (6, 23, 24) may be attributed to their high permeability to K^+^ (19, 25–28) and the function of the T-type channel Ca_v_3.2. The channel’s subunit α_1H_ is expressed in rat, murine, and bovine glomerulosa cell (29, 30). In view of the very negative membrane potential of isolated glomerulosa cells (27, 31), basal Ca^2+^ influx was attributed to a steady-state window current (19, 32). The control of Ca_v_3.2 in glomerulosa cells has recently been analyzed in murine adrenal slices (30), in which cells had a mean resting potential of −82 mV. Spontaneous membrane potential oscillations generated by Ca_v_3.2 were observed between −87 and −75 mV. Increasing [K^+^] up to 5 mM depolarized the membrane and increased oscillation frequency and peak amplitudes, whereas the increased frequency upon AII stimulation was most probably due to a G_i_-mediated shift in the voltage dependence of channel activation toward more negative potentials (33). In either cases, the ensuing Ca^2+^ signal (*via* CaMKII and p42/44 MAP kinase) acts on hormone-sensitive lipase (34) and StAR (13, 35, 36) [similarly to the actions of PKA (37)].

Angiotensin II stimulates aldosterone secretion after binding to AT_1_ receptors (AT1Rs) (38–40). Acting *via* the G-protein G_q_ and phospholipase C_β_, it induces the formation of inositol 1,4,5-trisphosphate (IP_3_) (41–43) which, through specific receptors [IP3Rs (44–46)] generates Ca^2+^ signal. Out of the three receptor isoforms expressed in glomerulosa cells (47), the dominant IP_3_R1 exhibits the greatest affinity for IP_3_. The initial Ca^2+^ release is followed by Ca^2+^ influx (48, 49) through store-operated (50, 51) and later *via* T-type Ca^2+^ channels (18, 33) [but see Ref. (52)]. In isolated rat glomerulosa cells, AII-induced T-type current is activated by depolarization (19) brought about by the inhibition of the Na^+^/K^+^ pump (53) and by the two-pore domain K^+^ channel TASK (25, 54). In murine cells maintained *in situ*, T-current is enhanced by a G_i_-mediated increase in the frequency of oscillating action potentials (30, 33).

Angiotensin II inhibits L-type current (55) and thus attenuates Ca^2+^ signals evoked by high [K^+^] (56, 57). This effect of the peptide is mediated by the G-protein G_i_, expressed in glomerulosa cells (55, 58).

Due to space limits, this review does not deal with diacylglycerol – protein kinase C (PKC), lipoxygenase, and MAPK pathways [reviewed, e.g., in Ref. (6)].

### Signaling in Fasciculata Cells

The physiological stimulus of glucocorticoid synthesis and secretion by fasciculata cells is ACTH, acting *via* MC2R receptors and cAMP. The mode of cAMP action is identical to that described above for glomerulosa cell [for review, see Ref. (59)].

ACTH action on fasciculata cells requires Ca^2+^. As observed already in the 70s, ACTH induces membrane potential changes (60) due mainly to Ca^2+^ influx (61). Both T-type (Ca_v_3.2) and L-type (Ca_v_1.3 and a non-identified) isoforms were characterized in bovine fasciculata cells, and their participation in ACTH- and AII-stimulated cortisol secretion was demonstrated (62). We are not aware of data on Ca^2+^ channels in rat and native human fasciculata cells; however, the observation that rat fasciculata cells were unresponsive to 13 mM K^+^ (23) indicates the lack of T-type Ca^2+^ channels. The resting membrane potential in bovine fasciculata cells is set by the background K^+^ channel bTREK-1 (63), whereas the TASK-3 background K^+^ channel, characteristic for rat glomerulosa cell (26), is undetectable in bovine fasciculata cells (64).

Albeit AT1R is expressed in human, bovine, and ovine fasciculata cells (65–67) data whether AII *alone* stimulates cortisol secretion are conflicting (62, 68–70). Rat *fasciculata* cells do not express detectable amounts of AT1R (71–73) [but see Ref. (74)] or inositol 1,4,5-trisphosphate receptor (IP3R) mRNA (47) and, accordingly, AII does not stimulate steroid production in these cells (23, 74, 75).

The expression of AT1R in fasciculata cells and the stimulation of cortisol secretion by the peptide raise the question whether AII plays any role in the control of cortisol secretion in man. In lack of comprehensive studies, we hypothesize that in stress situations, stimulation of fasciculata cells by AII may contribute to the stimulatory action of ACTH. On the other hand, in case of long-term high AII levels, cortisol secretion is maintained at resting level by the feed-back control of ACTH secretion.

The human adrenocortical cancer-derived H295R cell, a widely used model for studying steroid production, does not express either MC2R receptors (76) or Ca_v_3.2 T-type Ca^2+^ channels (30) [but see Ref. (77)]. Not surprisingly, these cells are insensitive to ACTH and respond to K^+^ at supraphysiological concentrations only (78).

## Interaction of Signaling Pathways in Adrenocortical Cells

### (Auto)Regulation of Ca^2+^ Metabolism by Ca^2+^

The formation, metabolism, and the action of IP_3_ all depend on cytosolic [Ca^2+^] ([Ca^2+^]_c_). Phospholipase C_γ_, generating IP_3_ from PIP_2_ (79) and the IP_3_ metabolizing IP_3_-3 kinase are both activated by Ca^2+^ (80–82). High [Ca^2+^]_c_ may reduce IP_3_ binding (83), whereas elevation of [Ca^2+^]_c_ up to ~300 nM increases the sensitivity of IP_3_R1 to IP_3_ [reviewed in Ref. (6)]. These characteristics play an important role in the oscillatory pattern of Ca^2+^ release. IP_3_R phosphorylation by PKA, PKC, or CaMKII enhances Ca^2+^ release, while calcineurin decreases this phosphorylation state (84). Also, calcium–calmodulin activates the plasmalemmal Ca ATPase (85) and inhibits the Na^+^/K^+^ ATPase (86, 87), the latter resulting in depolarization and Ca^2+^ influx through T-type channels (see Signaling Pathways in Glomerulosa Cells).

### Effects of Ca^2+^ on Cytosolic cAMP

Early reports on K^+^-evoked cAMP formation suggested a role for Ca^2+^ in the activation of AC (88, 89). Maintained secretagogue effect of ACTH in rat glomerulosa (90) and bovine fasciculata cells is also Ca^2+^-dependent (62) with calcium–calmodulin affecting primarily the formation of cAMP (91). In fact, in bovine cells, the effect of ACTH on cAMP formation correlates to extracellular [Ca^2+^] (92), and ACTH-induced cAMP formation is potentiated by AII in the presence of Ca^2+^ only (69).

Nevertheless, conflicting data were reported concerning the effect of AII on cAMP formation in bovine adrenocortical cells (69, 93–95). Reduced cAMP formation was reported in AII-stimulated rat glomerulosa cells (96, 97), whereas enhanced cAMP formation was observed in the human H295R cell (98). In this respect, the Ca^2+^ sensitivity of different transmembrane adenylyl cyclase isoforms (99, 100) should be considered. The Ca^2+^-activatable isoform AC1 is expressed in human glomerulosa and fasciculata cells (99); the Ca^2+^/calmodulin-activatable AC3 was found in human (99), rat (101), and bovine (69) glomerulosa cells. Ca^2+^-inhibited isoforms (AC5 and AC6) were detected in human (99) and rat glomerulosa cells (102). It should also be kept in mind that Ca^2+^-activatable AC isoforms are more responsive to store-operated Ca^2+^ entry than to Ca^2+^ release. This phenomenon is due to the colocalization of Ca^2+^ activatable AC isoforms and store-operated Ca^2+^ channels in plasmalemmal lipid rafts (103) and may account for the delayed cAMP response to AII (98).

After the description of G_i_ in rat glomerulosa cells (58), the reported inhibition of AC by AII was attributed to this inhibitory G-protein (55, 95). Summarizing, the cell-type differences in the effect AII on AC may be attributed to G_i_ density and the ratio of the various AC isoforms.

**Figure 1:**
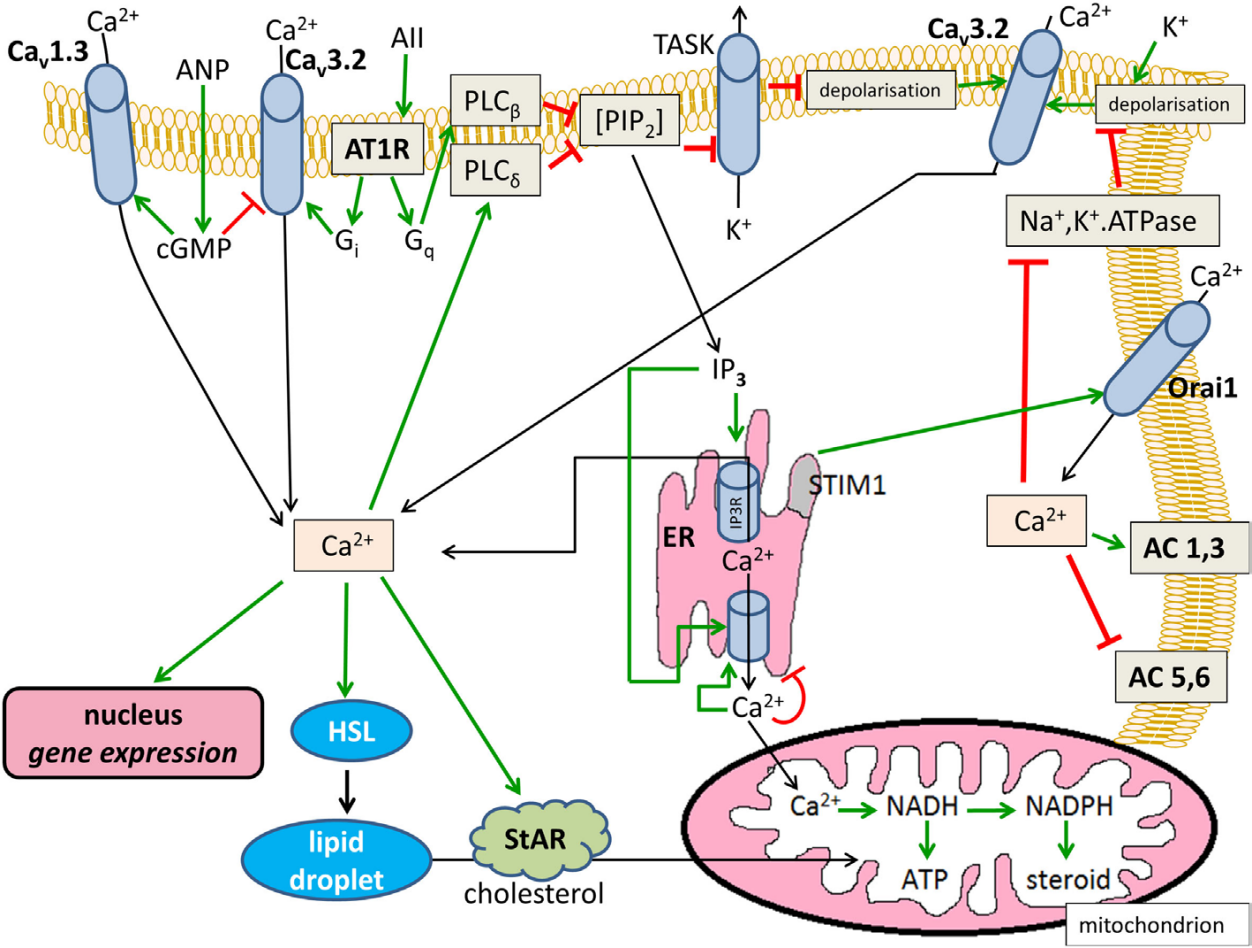
**Effects of Ca^2+^ on cytosolic cAMP in glomerulosa cells**. Positive modulations are shown with green arrows and negative effects are shown with red blunted arrows. Black arrows indicate substance transport. Ca_v_1.3, L-type voltage-dependent Ca^2+^ channel; Ca_v_3.2, T-type voltage-dependent Ca^2+^ channel; ANP, atrial natriuretic peptide; AII, angiotensin II; AT1R, angiotensin II receptor type 1, G_i_ and G_q_, heterotrimeric G-proteins; PIP_2_, phosphatidyl inositol 1,4,5-trisphosphate; TASK, KCNK3 or KCNK9-type K^+^ channel; AC, transmembrane adenylyl cyclase; ER, endoplasmic reticulum; IP_3_, inositol 1,4,5-trisphosphate; IP3R, IP_3_ receptor; HSL, hormone-sensitive lipase; StAR, steroidogenic acute regulatory protein.

The Ca^2+^-modified signaling pathways are summarized in Figure 1.

### Effects of cAMP on Ca^2+^ Signaling

ACTH or cell-permeable cAMP analogs may induce a sustained Ca^2+^ signal after a lag time of a few minutes as was shown in rat (104), bovine (92), and human glomerulosa cells (105), as well as in H295R cells (106). Several molecular interactions may warrant such an effect. PKA phosphorylates L-type Ca^2+^ channels (105, 107) [but see Ref. (108)], the ensuing Ca^2+^ current activates phospholipase C_δ_, and the generated IP_3_ induces Ca^2+^ release from the endoplasmic reticulum (ER). In addition, PKA also phosphorylates and activates IP_3_R1 [reviewed in Ref. (6)]. In fact, ACTH evokes a small phosphoinositide response (109) and PKC activation (110) in rat glomerulosa cells. By the same token, 8Br-cAMP enhanced AII-induced IP_3_ formation in bovine cells (111). Taken together, cAMP and its downstream effectors may enhance both Ca^2+^ influx and Ca^2+^ release in the adrenals.

**Figure 2:**
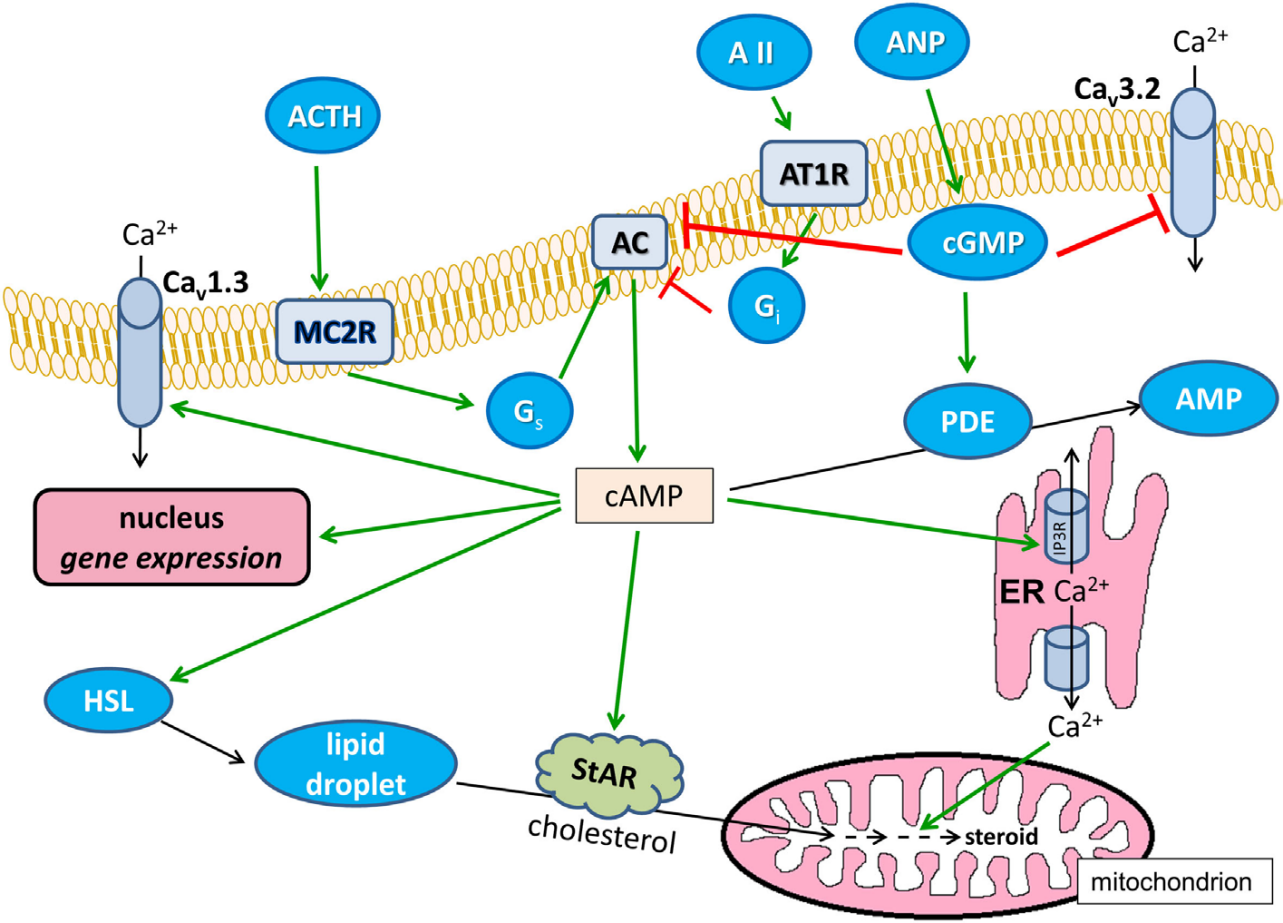
**Effects of cAMP on Ca^2+^ signaling in glomerulosa cells**. Positive modulations are shown with green arrows and negative effects are shown with red blunted arrows. Black arrows indicate substance transport. Ca_v_1.3, L-type voltage-dependent Ca^2+^ channel; Ca_v_3.2, T-type voltage-dependent Ca^2+^ channel; ANP, atrial natriuretic peptide; AII, angiotensin II; AT1R, angiotensin II receptor type 1, G_i_ and G_q_, heterotrimeric G-proteins; AC, transmembrane adenylyl cyclase; ER, endoplasmic reticulum; IP3R, IP_3_ receptor; HSL, hormone-sensitive lipase; StAR, steroidogenic acute regulatory protein; ACTH, corticotropin; MC2R, melanocortin receptor type 2, PDE, cAMP phosphodiesterase; AMP, adenosine monophosphate.

The cAMP-modified signaling pathways are summarized in Figure 2.

### Signaling Modulation by cGMP

Cyclic GMP is formed after the activation of ANP receptors. The nucleotide reduces T-type Ca^2+^ current (112) and inhibits the AC (113). In addition, cGMP activates PDE2A (114–116), one of phosphodiesterase isozymes identified in adrenocortical cells [reviewed by Vezzosi and Bertherat (117)]. By these actions, ANP reduces basal and stimulated aldosterone secretion (112).

### Synergistic Effects of Ca^2+^ Release and Ca^2+^ Influx

Moderate hyperkalemia increases the sensitivity and the maximal aldosterone response to AII (118–120). Potassium (4–8 mM) potentiates the secretory response to thapsigargin (evoking net Ca^2+^ release from the ER), whereas the secretory effects of thapsigargin and AII (300 pM) are additive only (119). When net Ca^2+^ release was induced with Ni^2+^, an inhibitor of microsomal Ca^2+^ uptake, the aldosterone response to physiological concentrations of K^+^ was again potentiated (121). These observations indicate that Ca^2+^ release and influx act in synergism on aldosterone secretion.

The synergism between Ca^2+^ release and influx may be explained by the formation of microdomains. Increased subplasmalemmal [Ca^2+^] (formed around the orifice of Ca^2+^ channels) may activate e.g., Ca^2+^-dependent AC isoforms and may induce specific gene expression (122, 123). On the other hand, Ca^2+^ release into the perinuclear space may turn on Ca^2+^-dependent nuclear genes and enhance NAD(P)H formation in ER-vicinal mitochondria (see Ca^2+^ Signal and Mitochondrial Function). In addition, the reduction in exchangeable Ca^2+^ pool during exposure to AII (124) may be counterbalanced by concomitant Ca^2+^ influx.

Angiotensin II-induced initial IP_3_ peak is followed by sustained suprabasal IP3 formation (41). Li^+^ inhibits the resynthesis of phosphoinositides and precludes the maintained formation of IP_3_, and thus attenuates the post-initial phase of AII-induced (but not ACTH-induced) aldosterone output of glomerulosa cells (125). This indicates that sustained suprabasal IP_3_ formation, Ca^2+^ release and, probably, store-operated Ca^2+^ entry all support long-lasting aldosterone secretion.

## Effects of Convergent Signaling on Gene Expression

Both Ca^2+^ and cyclic nucleotide signaling affect the transcriptome of adrenocortical cells (126, 127). Complex transcriptional or epigenetic (128) changes during adrenal zonation, remodeling, and neoplastic transformation are beyond the scope of this study [for review, see, e.g., Ref. (129, 130)]. Instead, we focus on instances where gene expression is modulated by parallel signal transducing pathways. One illustrative example of such an interplay involves the transcriptional regulation of hormone-sensitive lipase and StAR, both of which are induced by the cAMP-PKA (12–14) and by the Ca^2+^ pathway (13, 34–36). Along similar lines, Ca^2+^ and cAMP, through overlapping *cis* regulatory elements, synergistically induce the transcription of aldosterone synthase (CYP11B2) (131) and (as observed in non-adrenal cells) the mitochondrial Ca^2+^ uniporter (MCU, see below) (132). Transcription of type I 3β-hydroxysteroid dehydrogenase (HSD3B1), expressed predominantly in the human zona glomerulosa (133), can be induced by AII but not by K^+^ (134). A plausible explanation is that only AII [in part *via* PKC (135, 136)] recruits the nuclear receptor subfamily 4 (NGFI-B), which is also necessary for the induction of HSD3B1. In contrast, both AII and K^+^ induce CYP11B2 expression *via* the Ca^2+^/CaMK and MAP kinase pathways (137).

In vascular smooth muscle cells, both G_s_- and G_q_-initiated signaling dampen the expression of AT1R through mRNA destabilization (138). Interestingly, to carry out this mRNA degradation, the pathways partially converge on PKA (139). An effect closely reminiscent of such a convergence was observed in H295R cells where forskolin, db-cAMP, and AII all brought about a rapid drop in AT1R message levels. [Nevertheless, long-term AT1R repression was induced with forskolin/db-cAMP only (140).]

## Ca^2+^ Signal and Mitochondrial Function

Calcium activates three dehydrogenases in suspended or homogenized mitochondria (141). Ca^2+^-dependent mitochondrial NADH and NADPH (NAD(P)H) formation in living cells was first demonstrated in K^+^-stimulated glomerulosa cells (142). Similar response to AII and vasopressin was also reported (143, 144). The significance of increased NADH and ensuing ATP production (145) in any biological response is obvious, whereas NADPH is a cofactor of steroid biosynthesis (1). Noteworthy, the spatial and temporal pattern of AII-induced cytosolic Ca^2+^ signal depends on mitochondrial metabolism (78).

The primary event in the mitochondrial response to a cytosolic Ca^2+^ signal is the transfer of the ion into the mitochondrial matrix (146–149). Ca^2+^ transport occurs through the MCU complex, the velocity of which is a sigmoid function of [Ca^2+^]_c_ due to the allosteric control of the MCU channel by the regulatory subunits MICU1 and MICU2 [reviewed in Ref. (150)]. IP_3_-induced Ca^2+^ release from the ER generates high-Ca^2+^ microdomains between the ER and mitochondria and allows for mitochondrial Ca^2+^ uptake by the low-Ca^2+^-affinity MCU (151). However, the mitochondria of glomerulosa cells are uniquely sensitive to Ca^2+^ (152) and influx-induced low-Ca^2+^ signals are also effective in elevating mitochondrial [Ca^2+^] ([Ca^2+^]_m_) (153). This responsiveness may be essential for maintained aldosterone secretion in response to long-lasting hyperkalemia, characterized by small elevation of [Ca^2+^]_c_.

Increased [Ca^2+^]_m_ and the ensuing NAD(P)H formation play an essential role in the stimulation of aldosterone production. Targeted mitochondrial expression of a Ca^2+^ binding protein reduces both [Ca^2+^]_m_ and NAD(P)H and ensuing aldosterone production in response to AII (154). The opposite, increased mitochondrial Ca^2+^ uptake after the knockdown of p38 MAPK or the silencing of mitochondrial protein OPA1 results in increased NAD(P)H formation and enhanced aldosterone production (155).

A recently recognized and biologically significant action of Ca^2+^ signaling is the formation of cAMP in mitochondria. In addition to the nine isoforms of transmembrane AC, a soluble isoform (sAC) was prepared from testis (156). Its activity is not influenced by forskolin or G-proteins but increased by bicarbonate (157) and Ca^2+^ (158). The expression of sAC in the mitochondrial matrix together with a degrading mechanism sensitive to phosphodiesterase 2A inhibitors were recently described in HeLa cells (159, 160). The activity of intramitochondrial sAC increased in response to mitochondrial Ca^2+^ signal in HeLa and CHO cells and in rat cardiomyocytes (161). Importantly, mitochondrial cAMP (mt-cAMP) supported ATP formation (160, 161).

The sAC is also expressed in H295R adrenocortical cells, and it is found in the particulate fraction predominantly. In these cells, AII-induced mitochondrial Ca^2+^ signal increased the formation of mt-cAMP, and this response was enhanced by the PDE2A inhibitor EHNA. Mitochondrial cAMP signaling was attenuated with the sAC inhibitor 2-OH-estradiol, after silencing of the sAC gene and by the buffering of mitochondrial Ca^2+^ by S100G protein. All these maneuvers also attenuated aldosterone production, showing the cell-type-specific significance of mt-cAMP for the first time (98).

## Conclusion

Adrenocortical steroid production is under the control of both Ca^2+^ signaling and cyclic nucleotide metabolism. Importantly, these intracellular pathways are rarely, or probably never, independent. As postulated by Berridge in 1975 (162), cAMP and Ca^2+^ signaling may be antagonistic or synergistic in nature and, as hopefully accentuated by this review, the adrenal cortex is no exception to this rule. As shown in Figures 1 and 2, the aforementioned signal transducing pathways have the potential to interact at a number of points and levels of signaling. However, it needs to be stressed that these potential interactions are not enforced all at once but, instead, may be limited temporally and spatially (e.g., to signaling microdomains). Also, significant variance in the expression pattern and intensity of the relevant signaling molecules is to be expected depending on species and on the stimuli the organism is concurrently exposed to.

In spite of the interspecies differences and of the incongruences in some experimental data, it is probably safe to conclude that an adequate biological response necessitates the intricate interplay of parallel signaling pathways. That is to say that, e.g., in glomerulosa cells, sustained aldosterone response evoked by long-lasting tonic stimuli will be satisfactory only if the Ca^2+^ release/influx is accompanied by the sufficient formation of cAMP. Albeit the secretagogues AII and K^+^ invoke predominantly Ca^2+^ signaling and the effects of ACTH are mediated chiefly by cAMP, the increase of both factors at the same time may *potentiate* the final response. Thus, the separate intracellular pathways need not be activated to the same extent but nevertheless have to be recruited simultaneously for a sufficient steroid production to follow.

## Author Contributions

AS and GS compiled the literary data and wrote the manuscript. Data on the control of expression have been discussed with LH.

## Conflict of Interest Statement

The authors declare that the research was conducted in the absence of any commercial or financial relationships that could be construed as a potential conflict of interest.
